# Economic Spectrofluorometric Bioanalysis of Empagliflozin in Rats' Plasma

**DOI:** 10.1155/2021/9983477

**Published:** 2021-05-11

**Authors:** Bassam Ayoub, Noha El Zahar, Haidy Michel, Mariam Tadros

**Affiliations:** ^1^Pharmaceutical Chemistry Department, Faculty of Pharmacy, The British University in Egypt, ElSherouk City, Cairo 11837, Egypt; ^2^The Center for Drug Research and Development (CDRD), Faculty of Pharmacy, The British University in Egypt, ElSherouk City, Cairo 11837, Egypt; ^3^Pharmaceutical Analytical Chemistry Department, Faculty of Pharmacy, Ain Shams University, Organization of African Unity Street, Abassia, Cairo 11566, Egypt; ^4^Medicinal Chemistry Department, Faculty of Pharmacy, King Salman International University, Eas-Sedr, South Sinai, Egypt; ^5^Pharmacology and Toxicology Department, Faculty of Pharmacy, Ain Shams University, Organization of African Unity Street, Abassia, Cairo 11566, Egypt

## Abstract

A simple, economic, green, and sensitive bioanalytical method for empagliflozin bioassay in rats' plasma was employed successfully owing to the empagliflozin native fluorescence behavior. Enhanced liquid-liquid extraction, using diethyl ether (DEE), was successfully employed for the improved extraction of empagliflozin from rats' plasma based on its high value of logP as 1.8 that boosted the drug migration from plasma to the organic layer. The relative fluorescence intensity for empagliflozin was recorded at emission (299.4 nm) after excitation at 226.5 nm. The method was validated with satisfactory results for linearity (500–5000 ng/mL), trueness, precision, the matrix effect, and extraction recovery. The matrix effect ranged between 15.63% and 23.10% for LQC and HQC samples, respectively. Extraction recovery ranged between 54.61% and 62.54% for LQC and HQC samples, respectively. Bias values for the trueness ranged between −10.62 and +14.95, while %RSD values for the precision ranged between 5.39% and 9.33%. The method was successfully applied to rats' plasma samples that included six rats, and the drug concentration was determined in their plasma after 1 hour (estimated Cmax based on literature) following oral administration of empagliflozin with a concentration of 10 mg/Kg, p.o.. The developed cost-effective spectrofluorimetric method in the present work will be of beneficial use in further pharmacokinetic studies that include rats' plasma and biological fluids. Moreover, with the suitable modifications, the described novel extraction of empagliflozin could be adopted to human plasma samples and future clinical studies. Moreover, development of new simple cost-effective methods is necessary to give the researchers a set of “varieties” that they can use according to the laboratory limitations, especially in the developing countries in addition of being a greener method due to the lower consumption of toxic solvents and lower waste production.

## 1. Introduction

Type 2 diabetes is increasing in predominance worldwide with its major serious complications despite the diverse therapeutic strategies. Furthermore, the foremost cause of morbidity and mortality for the type 2 diabetes patients is due to cardiovascular diseases [1, 2]. Sodium glucose linked transporter 2 inhibitors (SGLT2i) are cutting edge class of drugs for the management of type 2 diabetes proposing an original insulin-independent mechanism for refining the blood glucose levels [3, 4]. Empagliflozin (EFN) is one of the highly specific SGLT2i that induces glycosuria. It improves the blood glucose levels as an antidiabetic with promising HbA1c reduction results over the long term, better bodyweight control, and significant improvements in microvascular outcomes [5]. Moreover, EFN ameliorates endothelial dysfunction, reduces vascular damage and progression of heart failure, and suppresses atherogenesis with future considerations as a pluripotent drug [6, 7]. EFN, used as a monotherapy or add-on therapy to other antihyperglycaemic drugs, is well tolerated and rapidly absorbed following oral administration [8].

Many chromatographic methods with UV and mass detection were reported for the assay of EFN in pharmaceutical dosage form and biological fluids either alone or in combination with linagliptin, metformin, and/or other antidiabetics [9–26]. Yet, they lack of being simple, fast, and cost-effective approaches due to the required costly complex instrumentation and time-consuming multipart analytical procedures. In addition, a voltammetry method has been reported for the analysis and bioanalysis of EFN in pharmaceutical dosage form and human urine [27]. Spectrophotometric and chemometric methods were reported for EFN by the first author [28], but those methods missed the known higher sensitivity output of the spectrofluorometric analysis. The purpose of the current investigation is to provide a new greener bioanalytical method for the determination of EFN in rats' plasma with full detailed validation according to the FDA guidelines as an alternative for the high cost methods described in the literature with the possible reduced cost advantage.

Fluorescence is the most common and useful type of photoluminescence in analytical chemistry. Spectrofluorometric technique exhibits extremely high sensitivity, which can be applied to the detection of a very wide range of analytes in environmental and biological samples, especially in case of the native fluorescence of the drug without the need for the pretreatment derivatization or modification. Its capacity of detection is approximately one order of magnitude greater than that of molecular absorption spectroscopy, and its selectivity is clearly greater than that of other spectroscopic methods. Accordingly, spectrofluorometry has been applied for the analysis of different analytes in complex matrices [29–31]. Furthermore, in the developing countries (that include Egypt), the application of spectrofluorometry in bioanalysis continues to be popular due to the simplicity, the common availability of the instrumentation, and the low cost without the need of costly infrastructure that is not applicable in QC labs with limited fund.

**Figure 1:**
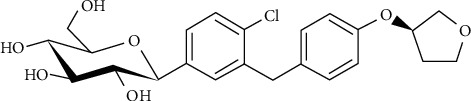
Chemical structure of empagliflozin (EFN).

Although two spectrofluorometric analytical procedures were described for EFN [32, 33], one of them was based on EFN derivatization before its determination in human plasma [32], while the other synchronous method did not consider its application to biological fluids and/or rats' plasma samples [33]. That defines the current study as the “first” spectrofluorometric method for EFN bioanalysis in rats' plasma based on its native fluorescence. In the current investigation, a simple spectrofluorometric method was described for the accurate and precise determination of EFN (Figure 1) in rats' plasma. FDA guidelines [34] were applied for the whole method development and bioanalytical validation including selectivity, linearity, trueness, interday and intraday precision, dilution integrity, extraction recovery, the matrix effect, and stability studies. This study will be of interest for pharmaceutical industry and researchers working in the QC area of pharmaceutical analysis and bioanalysis.

## 2. Materials and Methods

Shimadzu spectrofluorometer, RF-6000 model R928 photomultiplier, 3D measurement function, with 1 cm quartz cuvettes (Kyoto, Japan), controlled from a PC using the Lab Solution RF software for Windows (Rev.B.04.01, Shimadzu), was used. The temperature of the sample cuvette while taking measurement was left uncontrolled at room temperature. EFN certified to contain 99.7% and rats' plasma were “thankfully” supplied as a gift from the Center for Drug Research and Development (CDRD, the British University in Egypt). Analytical grade methanol was purchased from Sigma Aldrich (Germany).

Standard stock solution of EFN (1 mg/mL) was prepared in methanol; then, standard and quality control (QC) working solutions with different concentrations were prepared in methanol (100, 200, 400, 500, 600, 800, and 1000 *µ*g/mL). 10 *μ*L of each one of the prepared working solutions was used to spike 1990 *μ*L blank plasma to prepare calibration and QC samples with final concentrations of 500 (LQC), 1000, 2000, 2500 (MQC), 3000, 4000, and 5000 (HQC) ng/mL (for studying linearity, trueness, and precision).

### 2.1. Regarding Spiked and Real Samples Preparation

Two millilitres of rats' plasma was liquid-liquid extracted using 6 mL of diethyl ether (and the whole method was repeated with another 6 mL at the end), vortexed for 1 min, centrifuged for 30 min at 6000 rpm (2,817 × g), and freezed. Then, the upper organic DEE layer was separated and evaporated using a vacuum concentrator at 50°C for 20 min till dryness. The EFN extracted samples were then reconstituted with 2 mL of methanol, vortexed, and then transferred into the cuvette. The relative fluorescence intensity for EFN-extracted samples was recorded at *λ* emission (299.4 nm) after *λ* excitation at 226.5 nm. The biological samples were constantly maintained at −20°C until use, and the calibration and QC samples were freshly prepared every day.

### 2.2. Regarding the Animal Study

Six Sprague–Dawley rats received EFN (10 mg/Kg, p.o.) dissolved in saline and sodium lauryl sulphate as surfactant. It is worthy to mention that using sodium lauryl sulphate was necessary because of the high lipophilic nature of empagliflozin to ensure its dissolution efficiently. One hour later (estimated Cmax based on literature following oral administration in rats [14]), blood samples were collected from the retroorbital plexus and treated with anticoagulant (heparin at a final concentration of 1 U/mL and EDTA at a final concentration of 1 mg/mL). Plasma (2 mL) was separated by centrifugation at 1000 rpm (78 × g) for 10 min and then used for the assessment of the EFN level as described in the sample preparation. EFN dose was calculated based on reported doses and human to animal dose conversions with full consideration to the differences in the surface area between species [35]. This study was approved by the British University in Egypt Faculty of Pharmacy Ethical Committee (code: EX-2008, approved on 9 December 2020). The mentioned ethical committee is recognized by ENREC (Egyptian Network of Research Ethics Committees), http://www.enrec.org/directory. Finally, the extracted plasma samples were frozen and stored until use.

### 2.3. Regarding Bioanalytical Method Validation and Stability Studies

Bioanalytical method validation was achieved in agreement with the FDA guidelines. The selectivity of the method was evaluated by comparing the extracted EFN emission spectra at 500 ng/mL with blank plasma. Six different batches of blank rats' plasma (from 6 different rats) donated from CDRD-BUE were checked for interference as a measure of selectivity. The linearity was detected using calibration standard samples within the range of 500–5000 ng/mL. The relative fluorescence intensity was used against concentrations to predict the calibration curve and the regression equation. EFN concentrations were calculated using the corresponding regression equation to check the trueness of the method. The trueness of the method denoted the closeness of a measured value to the actual one and was estimated as recovery percent (*R*%). To check the precision displaying the closeness of multiple samples measurements exhibiting the same concentration, QC samples were assayed three times (*n* = 3) within the same day to assess the intraday precision, while interday precision was assessed on three successive days (*n* = 3). Both intraday and interday percent relative standard deviations (%RSD) were calculated. Moreover, the effect of the matrix on the response was computed as the ratio of the relative fluorescence intensities arisen from recording the spectra of postpreparation EFN-spiked samples to that of the corresponding neat solutions. The extraction recovery was assessed from the ratio of the relative fluorescence intensities arisen from recording the spectra EFN spiked samples to the postpreparation spiked samples.

Evaluating the small but deliberate changes that could occur during the routine analysis was carried out through the assessment of the method robustness. Robustness was checked at 500 ng/mL through a slight variation in *λ* excitation at 226 nm and then by %RSD calculation. The dilution integrity experiment for the rats' plasma method was carried out at 5 times and 10 times dilution of the high concentration samples (10 *μ*g/mL), and the corresponding concentrations were calculated. The used high concentration was higher than the upper limit of quantification. The percentage change from the comparison sample should be within ±15%. Stability of LQC and HQC was estimated based on 4 different bioassays after leaving the samples 3 h in the cuvette or leaving them 3 h at room temperature (bench top short-term stability) or analyzing them after three complete cycles (each cycle for two weeks) of both freeze and thaw. Finally, stability for the long-term stability was investigated by checking the samples after two weeks while freezing at −80°C. The four adopted stability studies were conducted as per FDA guidelines.

## 3. Results and Discussion

**Figure 2:**
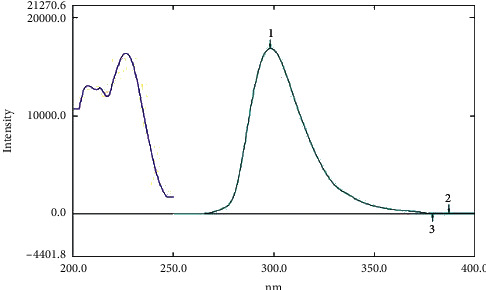
Fluorescence intensity for neat sample of EFN (750 ng/mL) in methanol at emission (299.4 nm) after excitation at 226.5 nm.

First, working on bioanalysis of recently approved antidiabetics provides valuable information about the behavior of the drugs in biological fluids either plasma samples or the other biological tissues such as brain tissue and/or urine samples. More applications could be developed using such information (like logP and enhanced extraction, for example) with outstanding outcomes up to even the repurposing of the antidiabetic drug for other uses in case of BBB crossing or other valuable behavior in biological fluids [36]. The proposed spectrofluorometric method was effectively applied for the determination of EFN in rats' plasma. To achieve the highest sensitivity and the finest peak shape, different parameters were raised. Upon a 3D scan, wavelengths were denoted, and the emission spectrum was obtained by scanning the emission monochromator at various *λ* emission, at a particular *λ* excitation. Eventually, the fluorescence intensity for EFN was best recorded at *λ* emission (299.4 nm) after *λ* excitation at 226.5 nm as shown in Figure 2. The scan speed, data interval, and the excitation and emission bandwidths were 600 nm/min, 0.2, and 15 nm, respectively. Moreover, among several tested diluting solvents, methanol showed the utmost relative fluorescence intensities [33].

Sample preparation had been enhanced to accomplish adequate recovery and the matrix effect. Studying EFN polarity and XlogP3-AA (=2) [37], protein precipitation and liquid-liquid extraction were evaluated for the plasma extraction. However, protein precipitation did not achieve the anticipated recoveries. Protein precipitation reported drawbacks [38], such as the long time taken for the evaporation and dryness phases, the quiet presence of remaining impurities in the samples, and the further needed clean-up, were inevitable in this study. While implementing the preliminary investigations with the current spectrofluorometric approach, protein precipitation using acetonitrile (4 times the plasma volume) showed inaccurate determinations. Many nonlinear results and even interference from blank plasma samples were noticed which may be attributed to the EFN high logP value. Moreover, using perchloric acid as the precipitating agent also failed to show satisfying results.

Liquid-liquid extraction was successfully applied for EFN plasma extraction achieving the best sensitivity, trueness, and recovery. It was assessed with different solvents such as ethyl acetate, DEE, and hexane and after the addition of different pH ranges buffers. Phosphate buffer (0.2 M, pH 3–10), acetate buffer (0.2 M, pH 3–5), and borate buffer (0.2 M, pH 8–10) solutions were used with the different mentioned extracting solvents in a trial to enhance the extraction procedure. Finally, the lower limit of quantification (LLOQ) was identified using the DEE that provided the cleanest extract and the highest recovery, while the other solvents showed lower efficiency to extract EFN (using the spectrofluorometric approach), while no difference was noticed upon the buffer addition. Using ethyl acetate and hexane showed the matrix effect of 25% and 28%, respectively, and both are higher than the average matrix effect (19.4%) received from DEE. In addition, both ethyl acetate and hexane showed recoveries below 50%, so they were excluded from the extraction parameters, especially no significant difference was detected (2–4%) after the addition of different buffers (either phosphate or acetate or borate). Consequently, liquid-liquid extraction, using DEE, was successfully used for the enhanced extraction of EFN from rats' plasma based on its high value of logP as 1.8 that enhanced the drug migration from plasma to the organic layer.

The relative fluorescence intensity for EFN was recorded at *λ* emission (299.4 nm) after *λ* excitation at 226.5 nm. Spectrofluorometric bioanalysis provides major advantages over all the chromatographic methods in the literature that include simplicity and the lack of need to prepare the stationary and mobile phases which is time consuming and costly. Moreover, development of new simple cost-effective methods is necessary to give the researchers a set of “varieties” that they can use according to the laboratory limitations, especially in the developing countries in addition of being a greener method due to the lower consumption of toxic solvents and lower waste production.

**Figure 3:**
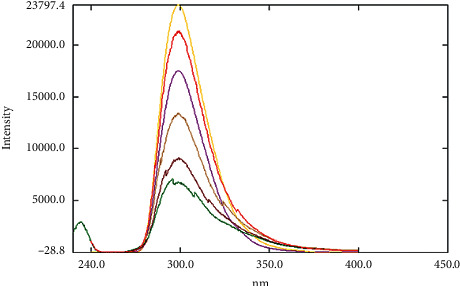
Overlay of EFN calibrators (500–5000 ng/mL) at emission (299.4 nm) after excitation at 226.5 nm before subtracting the blank fluorescence intensity to calculate the relative fluorescence intensity for all samples.

**Figure 4:**
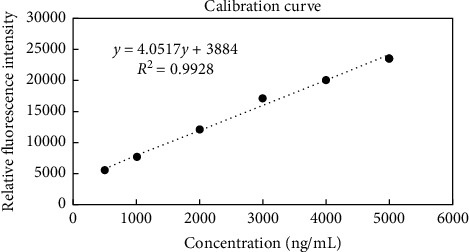
EFN calibrators (500–5000 ng/mL) at emission (299.4 nm) after excitation at 226.5 nm after subtracting the blank fluorescence intensity to calculate the relative fluorescence intensity for all samples.

The developed cost-effective spectrofluorimetric method in the present work will be of beneficial use in further pharmacokinetic studies that include rats' plasma and biological fluids. Moreover, with the suitable modifications, the described novel extraction of EFN can be adopted to human plasma samples and future clinical studies. The developed assay was validated efficaciously according to the FDA guidelines [29]. Good linearity and results were obtained as shown in consecutive increase in the fluorescence intensity (Figure 3) before subtracting the blank value of the blank plasma to calculate the relative fluorescence intensity and as per the following findings. The regression equation was computed and found to be RFI = 4.0517 Conc. +3884, *r*^2^ = 0.9928, and *r* = 0.9964, where RFI is the relative fluorescence intensity, *r*^2^ is the regression coefficient, and *r* is the correlation coefficient (Figure 4). LLOQ was found to be 500 ng/mL experimentally.

The method was validated with satisfactory results enclosing linearity range (500–5000 ng/mL), trueness, precision, the matrix effect, and extraction recovery. Relative bias values for the trueness (relative difference than 100% recovery) ranged between −10.62 and +11.70 for LQC, −7.4 and +5.69 for MQC, and 11.11 and 14.95 for HQC samples. The percent relative standard deviation (% R.S.D.) values for the precision of QC samples ranged between 5.83% and 9.33% for the interday repeatability, while they ranged between 5.39% and 6.85% for the intraday repeatability. Extraction recovery ranged between 54.61% and 62.54% for LQC and HQC samples, respectively, and the low values of the extraction recoveries may be due to the reported high binding of EFN to plasma proteins. Dilution integrity showed satisfying recoveries either for the five times or ten times dilution samples with bias below 5%, and all stability samples showed recoveries more than 85%, and all these results are compatible with FDA guidelines.

**Figure 5:**
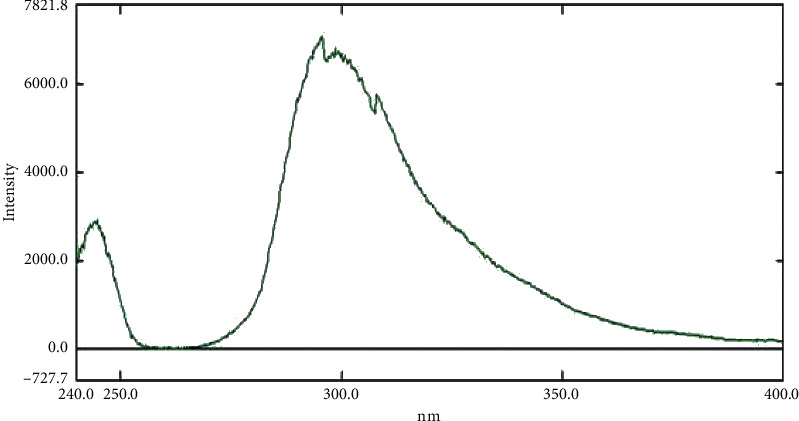
Emission spectra of a biological sample at 299.4 nm after excitation at 226.5 nm after 1 hour (estimated Cmax based on literature [14]) after oral administration of empagliflozin with a concentration of 10 mg/kg.

The influence of the matrix on the response was evaluated, and the matrix effect was consistent and ranged between 15.63% and 23.10% for LQC and HQC samples, respectively. Robustness was analyzed at 500 ng/mL with a slight variation in *λ* excitation at 226 nm, and %RSD showed no significant difference. The low value of %RSD reveals the effective reliability, consistency, and robustness of the adopted method. The method was successfully applied to biological samples that included six rats, and plasma EFN concentration was determined one hour (estimated Cmax based on literature [14]) after EFN administration (10 mg/Kg, p.o.). The mean and S.D. of the biological samples' concentration were 625.39 ng/mL and 262.48, respectively (Figure 5). The calculated mean concentration in biological samples was found within the developed calibration curve from the described underlying investigation.

## 4. Conclusions

The simple validated and robust spectrofluorometric method was proved to be suitable for EFN determination in rats' plasma using a one-step liquid-liquid extraction. The developed cost-effective bioanalytical method in the present work disclosed good precision, trueness, and linearity, which will be of beneficial use in further pharmacokinetic and preclinical studies. Spectrofluorometric bioanalysis provides major advantages over all the chromatographic methods in the literature that include simplicity and the lack of need to prepare the stationary and mobile phases which is time consuming and costly. Moreover, development of new simple and cost-effective methods is necessary to give the researchers a set of “varieties” to be used according to the laboratory limitations especially in the developing countries in addition of being a greener method due to the lower consumption of toxic solvents and lower waste production.

## Data Availability

The data (including figures) used to support the findings of this study are included within the article.

## Additional Points

Samples of empagliflozin are available from the CDRD Research Center.
